# Signal Propagation between Neuronal Populations Controlled by Micropatterning

**DOI:** 10.3389/fbioe.2016.00046

**Published:** 2016-06-15

**Authors:** Jonas Albers, Andreas Offenhäusser

**Affiliations:** ^1^Institute of Complex Systems, Bioelectronics (ICS-8), Forschungszentrum Jülich GmbH, Jülich, Germany; ^2^Peter Grünberg Institute/Institute of Complex Systems, Bioelectronics (PGI-8/ICS-8), Forschungszentrum Jülich GmbH, Jülich, Germany

**Keywords:** axon guidance, neural network, calcium imaging, microcontact printing

## Abstract

The central nervous system consists of an unfathomable number of functional networks enabling highly sophisticated information processing. Guided neuronal growth with a well-defined connectivity and accompanying polarity is essential for the formation of these networks. To investigate how two-dimensional protein patterns influence neuronal outgrowth with respect to connectivity and functional polarity between adjacent populations of neurons, a microstructured model system was established. Exclusive cell growth on patterned substrates was achieved by transferring a mixture of poly-l-lysine and laminin to a cell-repellent glass surface by microcontact printing. Triangular structures with different opening angle, height, and width were chosen as a pattern to achieve network formation with defined behavior at the junction of adjacent structures. These patterns were populated with dissociated primary cortical embryonic rat neurons and investigated with respect to their impact on neuronal outgrowth by immunofluorescence analysis, as well as their functional connectivity by calcium imaging. Here, we present a highly reproducible technique to devise neuronal networks *in vitro* with a predefined connectivity induced by the design of the gateway. Daisy-chained neuronal networks with predefined connectivity and functional polarity were produced using the presented micropatterning method. Controlling the direction of signal propagation among populations of neurons provides insights to network communication and offers the chance to investigate more about learning processes in networks by external manipulation of cells and signal cascades.

## Introduction

Large populations of neurons have the ability to carry out multiple complex processes in parallel facilitated by the highly ordered architecture of the network. Setting up these intricate networks necessitates the control of precise wiring of neuronal circuits. Nowadays, the concept of neural microcircuits is widely accepted based on experimental and computational studies (Bastos et al., 2012; Kwan and Dan, 2012). These circuits form more complex networks termed macrocircuits that connect various brain regions. Understanding the connections between regions (macroconnectome) and of microcircuits within regions (microconnectome) is a key challenge. Monitoring every neuron’s input and output is crucial for understanding its function in these circuits but is technically impossible and does not allow to unravel the structure–function relationship (Feldt et al., 2012).

Establishing these intricate networks *in vitro* requires control of precise wiring of neuronal circuits. This implies guidance on a single-cell level and on the scale of small populations of neurons. As neuronal development can be influenced by numerous cues manipulating the outgrowth of individual cells, such as extracellular signaling proteins (Richards et al., 1997; Arimura and Kaibuchi, 2007) or intrinsic factors like centrosome position (de Anda et al., 2005). But also the prior orientation of the cytoskeleton impacts the direction of axonal outgrowth by tensions generated by microfilaments inside the axon (Baas and Ahmad, 2001; O’Toole et al., 2008, 2015; Suter and Miller, 2011; Roth et al., 2012).

A multiplicity of approaches has been used to modify cell growth and to control polarity of neuronal networks *in vitro*. An ideal approach would offer full control of neuronal outgrowth on both the single-cell level and on the network scale. The system would provide full accessibility for electrical, chemical, and optical stimulation and recordings. Furthermore, it would be suitable for low- and high-cell density culture with arbitrary network geometries. All developed approaches comply with at least some or most of these criteria, but fall short of fulfilling them all. The best way to establish high-density culture with axon separation and controllable connectivity between neighboring populations of neurons can be achieved with microfluidic devices (Peyrin et al., 2011; Millet and Gillette, 2012; Park et al., 2014; Verhulsel et al., 2014). The downside of these systems is their enclosed geometry, which forestalls intracellular recordings from any cell on the substrate and limits electrophysiological measurements to approaches like those of Jokinen et al. (2013). In-mold patterning methods, Feinerman et al. (2005, 2008) and Biancardo et al. (2008) overcome the limitation of accessibility for electrophysiological manipulation and maintain the topological influence. Propagation of neuronal activity with a predefined direction was shown on large high-density populations of neurons (Feinerman et al., 2005, 2008) on these substrates, while the control of individual cells and their behavior during development is limited. To adapt network geometry and single-cell position to external measurement devices (Faid et al., 2005; Charrier et al., 2010), more suitable approaches have been developed, including laser micropatterning (Scott et al., 2012), photolithographic patterning (Li and Ho, 2008; Wheeler and Brewer, 2010), and microcontact printing (Bernard et al., 1998; Roth et al., 2012). A high spatial resolution can be achieved with microcontact printing with a trustworthy reproducibility in a broad range of dimensions. Submicrometer structures can be printed to influence neurite outgrowth (Schwaab et al., 2013), as well as small networks with control of single-cell outgrowth (Mourzina et al., 2006a,b; Offenhäusser et al., 2007; Charrier et al., 2010; Fricke et al., 2011; Roth et al., 2012; Yamamoto et al., 2012), and generate patterns spanning square centimeters of substrate area. Microcontact printing is even suitable for the formation of structures with dimensions of several hundred micrometers with a distinct network geometry (Albers et al., 2015). To the best of our knowledge, it has not yet been reported that directionality of signal propagation between adjacent neuronal populations can be controlled by the method of microcontact printing.

In the present study, we used a previously published method (Albers et al., 2015) to create daisy-chained populations of neurons with a triangular geometry. This allows controlled outgrowth of axons toward a predefined gateway between adjacent structures, where the base of the upper structure and the tip of the lower structure meets. Triangular-shaped networks of embryonic cortical rat neurons were produced by using microcontact printing to transfer patterns of substrate-bound proteins consisting of a mixture of poly-l-lysine and laminin. We presume that when encountered, the borders of protein structures lead to turning events in neurite outgrowth, as has been shown by Turney and Bridgman (2005) and thus, directional neuronal outgrowth can be induced. In this survey, we focus on the impact of neuronal outgrowth induced by the protein pattern geometry, the connectivity between adjacent populations of neurons, and the resulting direction of signal propagation among populations. Therefore, embryonic cortical rat neurons were cultured on patterned glass substrates up to 24 days *in vitro* (DIV). Subsequently, spontaneous neuronal activity was recorded optically and analyzed with a MATLAB script. Thus, we have a powerful tool to investigate network communication and information processing within and among networks of neurons *in vitro*. The beauty of this method is the full accessibility of single neurons for electrical and optical stimulation and recording during measurements with cellular resolution.

## Materials and Methods

### Stamp Production

A dark-field chrome mask with the triangular structures was written by an electron beam writer. A 5- to 12-μm thick layer of photoresist (AZ 4562, Clarion GmbH, Germany) was spin coated on a dehydrated 0.6 mm thick silicon wafer (5″ diameter, MEMC Electronic Materials, USA) to fabricate a mold for stamp production. The resist was dried for 60 s at 130°C. Subsequently, the structures were transferred using UV-photolithography, and the wafer was baked for 90 s at 140°C. MF-24-A (Karl Süss GmbH, Germany) was used for 50 s to develop the resist, and the reaction was stopped by washing with Milli-Q water. To transfer the structures 4.5 μm deep into the wafer, deep reactive ion etching with SF6 at 150 W for 10 min was used. A layer of (Tridecafluoro-1,1,2,2-tetrahydrooctyl)trichlorosilane (FOTCS) (Sigma, Germany) was linked covalently to the surface of the mold as a release layer by a vapor deposition process (45 mbar for 1.5 h) in argon atmosphere. Microstamps consisting of polyolefin plastomer (POP) were fabricated by hot embossing, as described previously (Mrksich et al., 1997; Chang et al., 2001).

### Structure Design

**Figure 1:**
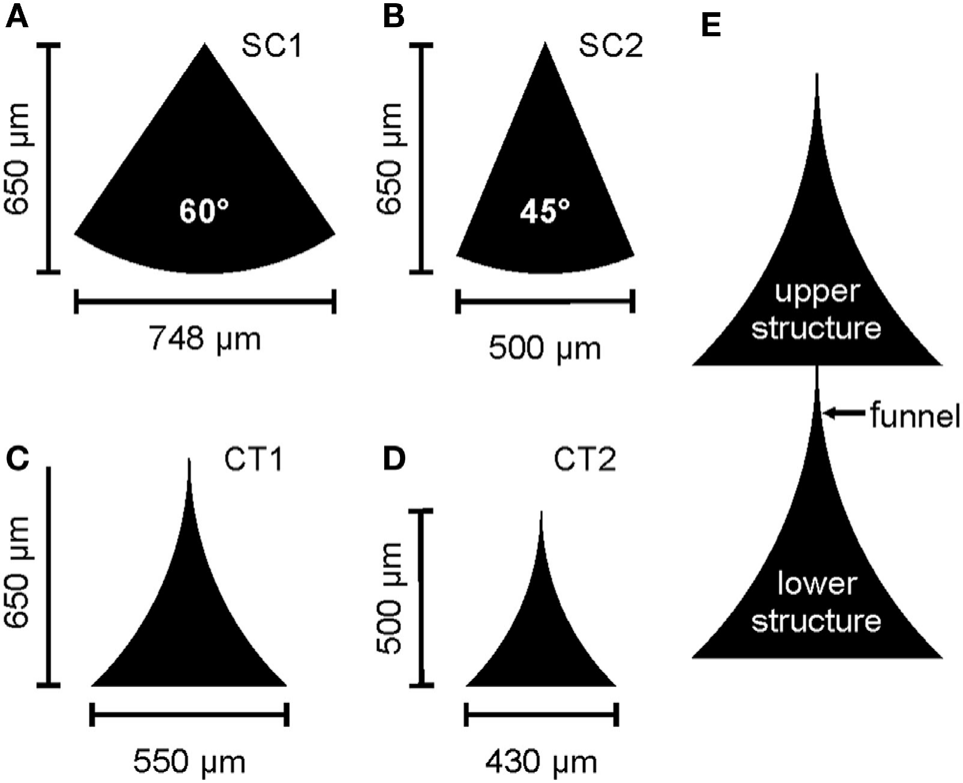
**Sketch of the different designs of the tested structures**. **(A,B)** A sector of a circle (SC) with different opening angles. **(C,D)** Curved triangular (CT) structures of different heights. **(E)** Section of the daisy-chained arrangement of a CT structure including the nomenclature used in the text for descriptions of the gateway. Reproduced with permission from Albers et al. (2015).

Daisy-chained lines of triangular structures of the same design were horizontally separated by 200 μm resulting in an array of 1 cm × 1 cm covered with structures. In this study, four different designs were used. Two sector of a circle (SC) with a height of 650 μm and an opening angle of 60° and 45°, “SC1” and “SC2,” respectively, and two differing triangles with curved sides (CT1 and CT2) were used (see Figure 1). The curved triangular (CT) structures were designed by subtracting two circles touching the base and the top of the triangle. CT1 used circles of a radius *r* = 900 μm and triangles of *w* = 550 μm and *h* = 650 μm, whereas CT2 used circles of a radius *r* = 700 μm and triangles of *w* = 430 μm and *h* = 500 μm. All structures were arranged in a way that the tip touches the base of the adjacent structure and forms a defined gateway between the structures (Albers et al., 2015).

### Sample Preparation

Microcontact printing (μCP) was used to transfer the protein structures onto a cell-repellent glass surface. This repellent behavior was achieved by silanizing with FOTCS at 45 mbar for 1.5 h in an argon atmosphere. Prior to stamping, the glass coverslips were sterilized with UV for 15 min, and the POP stamps were cleaned and sterilized in 70% ethanol in an ultrasonic bath for 15 min. To incubate the stamps in an inking solution [10 μg/mL FITC labeled poly-l-lysine (PLL) and 5 μg/mL laminin diluted in Gey’s Balanced Salt Solution (Sigma, Germany)], they were dried in a nitrogen stream and immersed in the solution structures face down. After 20 min of incubation, the stamps were entirely dried in a nitrogen stream and gently pressed onto the silanized glass coverslip for 2 min. The substrates were stored at 4°C in the dark prior to cell culture.

### Cell Culture

The preparation and culture conditions were previously described (Fricke et al., 2011). Briefly, primary cortical rat neurons from embryos of either sex were obtained from E18 Wistar rats and diluted in Neurobasal medium (Life Technologies GmbH, Germany) with 1% B-27 supplement (Life Technologies), 0.5 mM l-glutamine per hemisphere, and 50 μg/mL gentamicin. A cell concentration of 16,000 cells/cm^2^ was used for all cultures. The first media change after plating was performed after 3 h, and the entire medium was replaced. At subsequent media changes for every 3–4 days, half of the medium was exchanged. Cells were kept at 37°C, 5% CO_2_ and 100% humidity in the incubator.

### Immunofluorescence Analysis

Substrates for immunofluorescence analysis were cultured on 18 mm glass coverslips in a 12-well dish with the same cell concentration as for functional analysis. After 14 days, *in vitro* cells were rinsed thrice with preheated 1× phosphate buffered silane solution (PBS) prior to fixation with 4% paraformaldehyde in 1× PBS for 10 min at room temperature (RT). Substrates were rinsed thrice with 1× PBS subsequently and permeabilized with 0.3% Triton X-100 (Sigma) in blocking buffer (2% bovine serum albumin and 2% heat-inactivated goat serum in 1× PBS) for 10 min at RT. Another three rinsing steps were performed before samples were blocked with blocking buffer at 4°C in the dark overnight. Samples were incubated with primary antibodies against microtubule-associated protein 2 (MAP2) (2 μg/mL, Milipore) and anti-200 kDa neurofilament heavy (NFH) (2 μg/mL, abcam) both diluted (1:500 and 1:2000) in blocking buffer for 2.5 h at RT in a wet and dark chamber. Substrates were washed thrice with 1× PBS and incubated with secondary antibodies (Alexa Fluor 633 and Alexa Fluor 546, Invitrogen) diluted in blocking buffer (1:500) for 1.5 h in a wet and dark chamber at RT. After finally rinsing once with PBS and twice with Milli-Q water, substrates were embedded in fluorescent mounting media (Dako) and dried over night before imaging. Images were acquired with a Zeiss Observer.Z1 equipped with a Zeiss Colibri system and a PCO.edge 5.5 sCMOS camera using the Zeiss ZEN software. The resulting images were manually analyzed with respect to axonal and dendritic growth at the gateway between adjacent structures.

### Calcium Imaging

After 14–24 days, *in vitro* spontaneous neuronal activity was optically recorded by calcium imaging. For the experiments, cells were rinsed three times with preheated extracellular patch solution (E-patch) and incubated with 4 μM Fluo-4 AM (Invitrogen) diluted in E-patch for 45 min in the dark at RT. The E-patch contains CaCl_2_ (2 mM), HEPES (10 mM), KCl (3 mM), MgCl_2_ (1 mM), and NaCl (120 mM), and the pH value of the solution was adjusted with 1M NaOH to 7.3. In case, the osmolarity of the culture medium exceeded the osmolarity of E-patch by more than 10 mOsmol/kg, the osmolarity of E-patch was adjusted to match the value of the medium directly before the experiment with d-(+)-Glucose (Sigma). After incubation with Fluo-4 AM, the substrate was rinsed twice with E-patch, and the final volume of 2 mL E-patch was added to the 35 mm Petri dish. The samples were imaged after an additional rest time of ~10 min in the dark at RT. A Zeiss Observer.Z1 equipped with a Zeiss Colibri system and a PCO.edge 5.5 sCMOS camera was used for sequence acquisition. Time sequences with a length of 15–30 s were recorded with the Zeiss ZEN blue software at an exposure time of 5 ms and a frame rate of 200 frames/s. To achieve this temporal resolution, a 2 × 2 binning and an image size of 512 × 480 pixels (1.30 μm/pixel) were chosen. For video analysis, the sequences were converted to AVI files.

### Sequence Analysis

**Figure 2:**
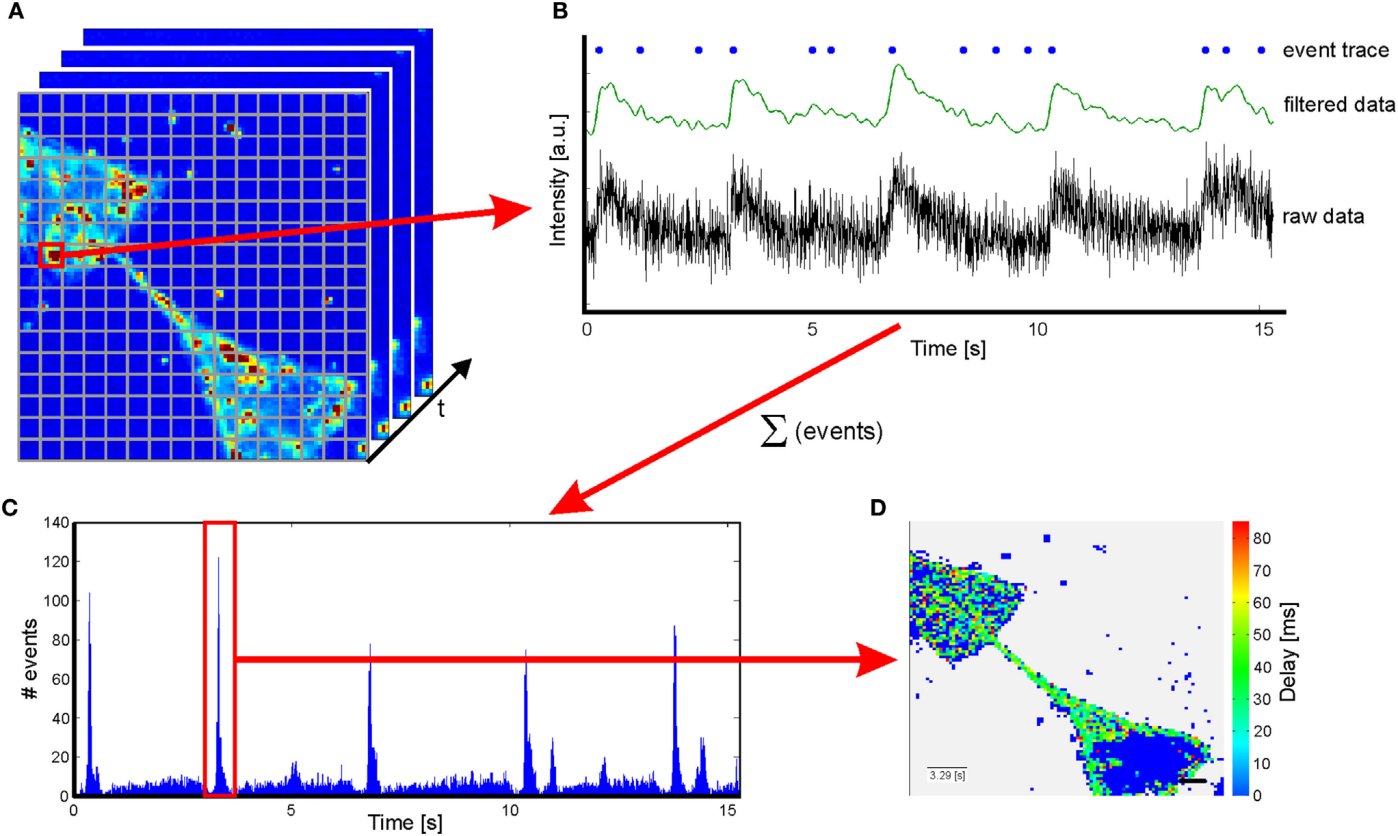
**Sketch of the script used to analyze sequences from calcium imaging**. **(A)** An additional binning is applied to obtain a resolution closer to cell dimensions. **(B)** Raw data are extracted from sequences and normalized prior to filter application. Increases in fluorescence intensity related to action potentials are identified by the first derivative of the filtered data and translated in an event traces. **(C)** A summation of the events over time results in a histogram enabling identification of substrate spanning excitation. **(D)** Color-coded delay plot of the substrate spanning excitation marked in red in the histogram in **(C)**. The insert in the lower left corner indicates the first event in the substrate spanning excitation. Scale bar: 50 μm.

The video sequences were analyzed with a script written in MATLAB. As the size of the 1.3 μm × 1.3 μm per pix in the recorded sequence is below single-cell dimensions, an additional binning of 5 × 5 pixels was applied (see Figure 2A). Thus, a resolution was achieved, matching with the dimensions of a cell body and which furthermore exhibits the benefit of reducing the calculation time by a reduction of dimensions. Intensity traces for each resulting pixel were extracted. Not all pixels within the frame correspond to a cell and thus exhibit changes in fluorescence intensity. As a consequence, potentially active traces have to be identified by their intensity change over time for visualization of the results and reduction computational resources. To differentiate active and non-active pixels, all sequences were normalized with a Gaussian filter with a window size of 45 frames. A decision criterion is defined by summing the mean intensity and its SD of the entire sequence: *A* = |mean_frame_ + std_frame_|. In addition, it is well known that the mean of a trace is stronger influenced by variations than the median of the same trace. As a consequence, a second decision criterion for individual traces is defined: *B* = |mean_trace_−median_trace_|. Here, *B* should be larger for traces with large variations in fluorescence intensity and the following is true: *B*_active_ > *B*_non-active_. Consequently, traces were considered as active if *A* × 0.95 < *B*. All other traces are defined as non-active and were used for background analysis. All potentially active and non-active traces were smoothed and normalized for time correlation analysis. For normalization, the following equation is applied: Δ*F* = (*F* − *F*_0_)/(*F*_max_ − *F*_0_), where *F* is the fluorescence intensity of the trace, *F*_0_ the minimal intensity, and *F*_max_ the maximal intensity of the sequence (see Figure 2B). At the same time, fluorescence intensity traces are smoothed with a Savitzky and Golay (1964) filter with polynomial order of 3 and a window size of 38 frames. To maintain the characteristics of the traces, the filter is applied from both sides (see Figure 2B). A smoothing of the traces is required, as the raw data traces exhibit a high flickering (see Figure 2B). For better comparison among one another, the fluorescence traces were translated into binary event traces with ones indicating a recognized action potential. Therefore, the background noise was approximated applying a histogram plot of the first derivative of the intensity of the non-active traces. By fitting a Gaussian distribution to the histogram, the sigma interval (SD) could be extracted. This sigma interval represents the background noise that was used for thresholding to identify peaks in potentially active traces. Events were identified from the first derivative of the active traces. Peaks higher than three times the SD of the background signal and with a minimal separation of 400 ms were counted as a single event (SE) associated with an action potential. The adapted activity and sigma thresholding for individual sequences enable us to identify intensity changes with a sensitivity of 8% intensity change in normalized traces. For the identification of substrate spanning excitation (SSE), event traces of all potentially active traces were accumulated in a histogram over time (see Figure 2C). Thus, SSE can be extracted from background signals using the region property method (MATLAB). The first excitation of each event trace during a SSE is used for delay analysis. SSE with a minimal temporal separation of 500 ms (first excitation to first excitation) can be identified. In a terminal step, a color-coded delay plot is generated for every SSE (see Figure 2D). The time of the first SE of the SSE is noted in the insert in the lower left corner, and a scale bar indicating 50 μm is inserted at the lower right corner according to the original pixel size. The resulting delay plots were manually analyzed based on their color coding.

## Results

For all experiments performed in this study, triangular protein structures as depicted in Figures 1A–D were transferred onto silanized glass coverslips by μCP, as previously described (Fricke et al., 2011; Wendeln and Ravoo, 2012; Albers et al., 2015). Cortical embryonic rat neurons were cultured on the substrates for 10–14 DIV for immunofluorescence analysis and 14–24 DIV for recordings of spontaneous activity by calcium imaging. The observed exclusive cell growth on the patterned structures, see Figure S1 in Supplementary Material, is mediated by the substrate-bound proteins. Nonetheless, it remained unclear how the geometry of protein structures impacts the connectivity between adjacent populations of neurons.

### Cell Growth

**Figure 3:**
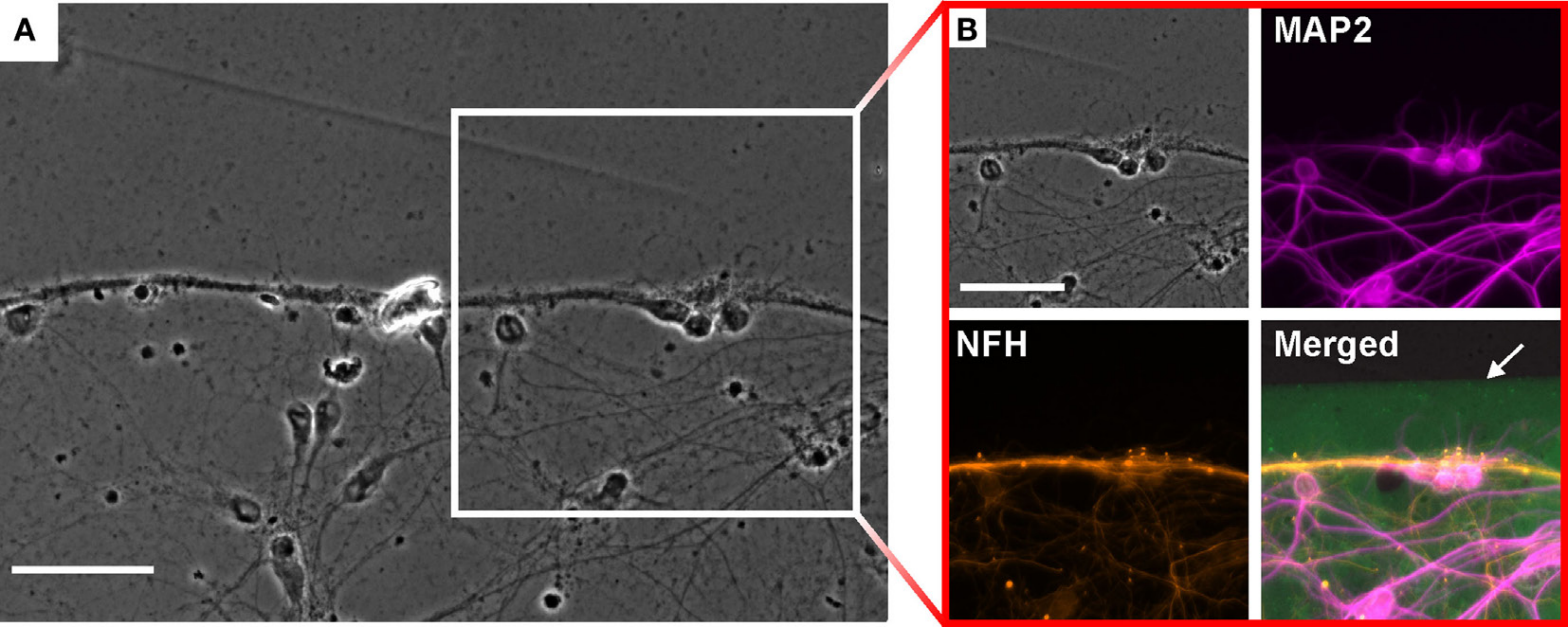
**Neuronal growth on the boundary of the structures after 14 days *in vitro***. **(A)** Phase contrast image of neurite growth on the edge of a protein structure. Scale bar: 50 μm. **(B)** Immunostained neurons for analysis of neurite growth close to the edge of the FITC (green) labeled protein. Axons [neurofilament heavy (NFH) antibody, orange] form a bundle parallel to the boundary, whereas the dendrites (MAP2, magenta) form a ramified network. The protein pattern is visualized by FITC labeling (green). Scale bar: 50 μm.

Immunofluorescence analysis was performed to characterize neurite growth induced by the protein structures. The formation of neurite bundles can be observed at the boundaries of the protein structures, while a ramified network of neurites is established inside the pattern (see Figure 3A; Figure S2 in Supplementary Material). The close-up in Figure 3B reveals the different components of the network. The dendrites, identified by MAP2 staining, form a uniform branched network without specific affinity to particular regions on the structure. In contrast, the axons, visualized by NFH staining, show the tendency to cluster in close proximity to the boundary of the protein structure. It is noticeable that the axon bundles are found with a distance ranging between 25 and 35 μm to the protein boundary. The dendrites, on the other hand, approach the boundary without any noticeable restrictions.

**Figure 4:**
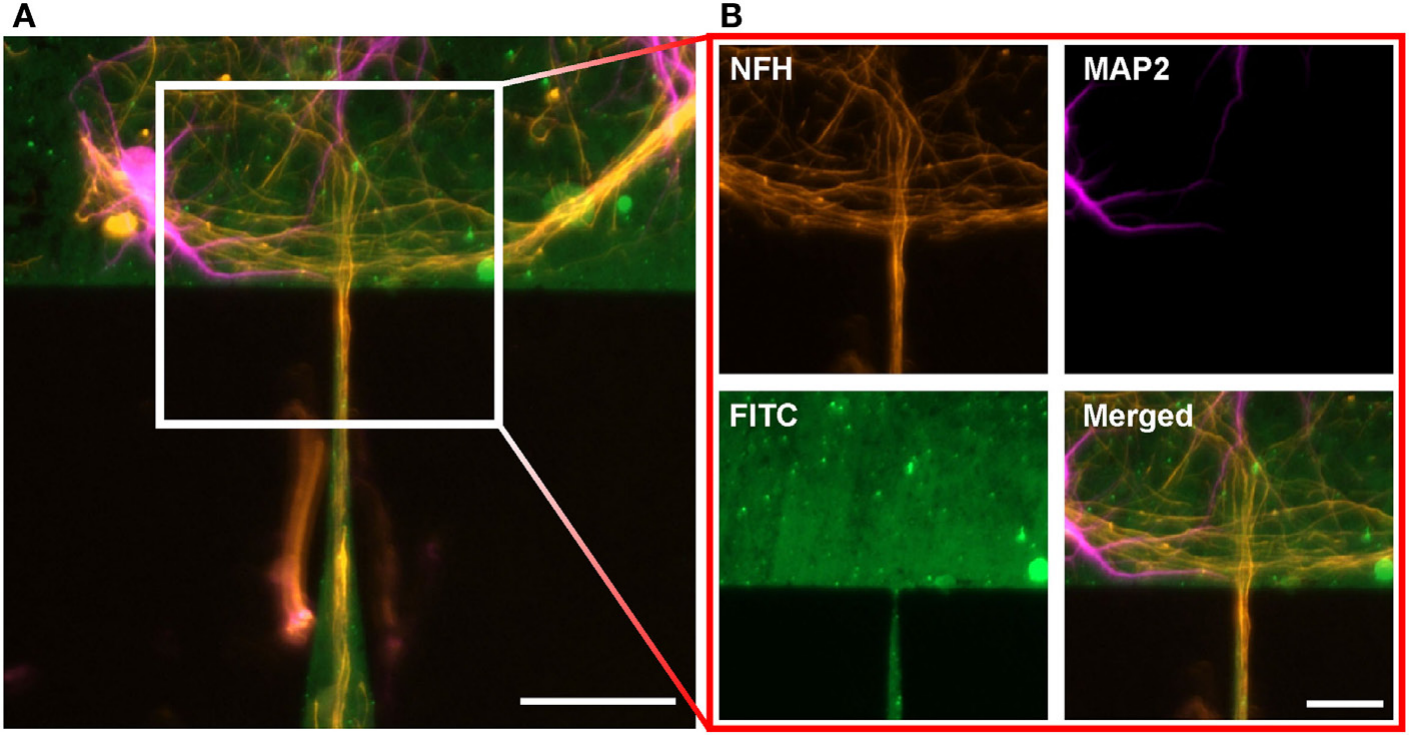
**Neuronal cell growth on the gateway between two adjacent structures after 14 days *in vitro***. **(A)** Cell growth between two CT1 structures. Neurons are stained against MAP2 (magenta) and NFH (orange). Scale bar: 50 μm. **(B)** Close-up of the gateway. The axons and the dendrites follow the base and overgrow the gateway without turning, whereas the axons from the lower structure sprout into the upper structure. Scale bar: 25 μm.

The situation changes when we focus on the gateway between adjacent structures. As on the edge of a protein structure, the formation of axon bundles can also be observed at the funnel where the lower structure transitions to the upper structure, for nomenclature see Figure 1E. The image of a CT1 structure in Figure 4A shows the structure of these bundles. In contrast to other boundary regions of the structure, a competing influence of two regions can be found. The fine structure of the tip from the lower structure acts like a funnel for the axon bundles, whereas the base of the upper structure shows similar behavior, as described above for the boundaries of protein structures. The bundles at the base follow the structure and overgrow the incoming tip without any change as a reaction to the entering axons (see Figure 4B) (NFH). The axons from the lower structure are funneled by the tip and sprout as a bundles into the upper structure. Here, the bundle roves and the single-axon strands interconnect with surrounding cells. Dendrites in the upper structure show a similar behavior at the gateway, as described for the axons without forming bundles (see Figure 4B) (MAP2). At the base of the upper structure, the dendrites follow the edge of the protein layer and approach the junction without changing the direction of growth. In contrast to the axons, a slight penetration of the incoming tip of the lower structure can be observed for dendrites directly at the junction. Interestingly, this does not necessarily lead to an incursion of the dendrite into the funnel.

**Figure 5:**
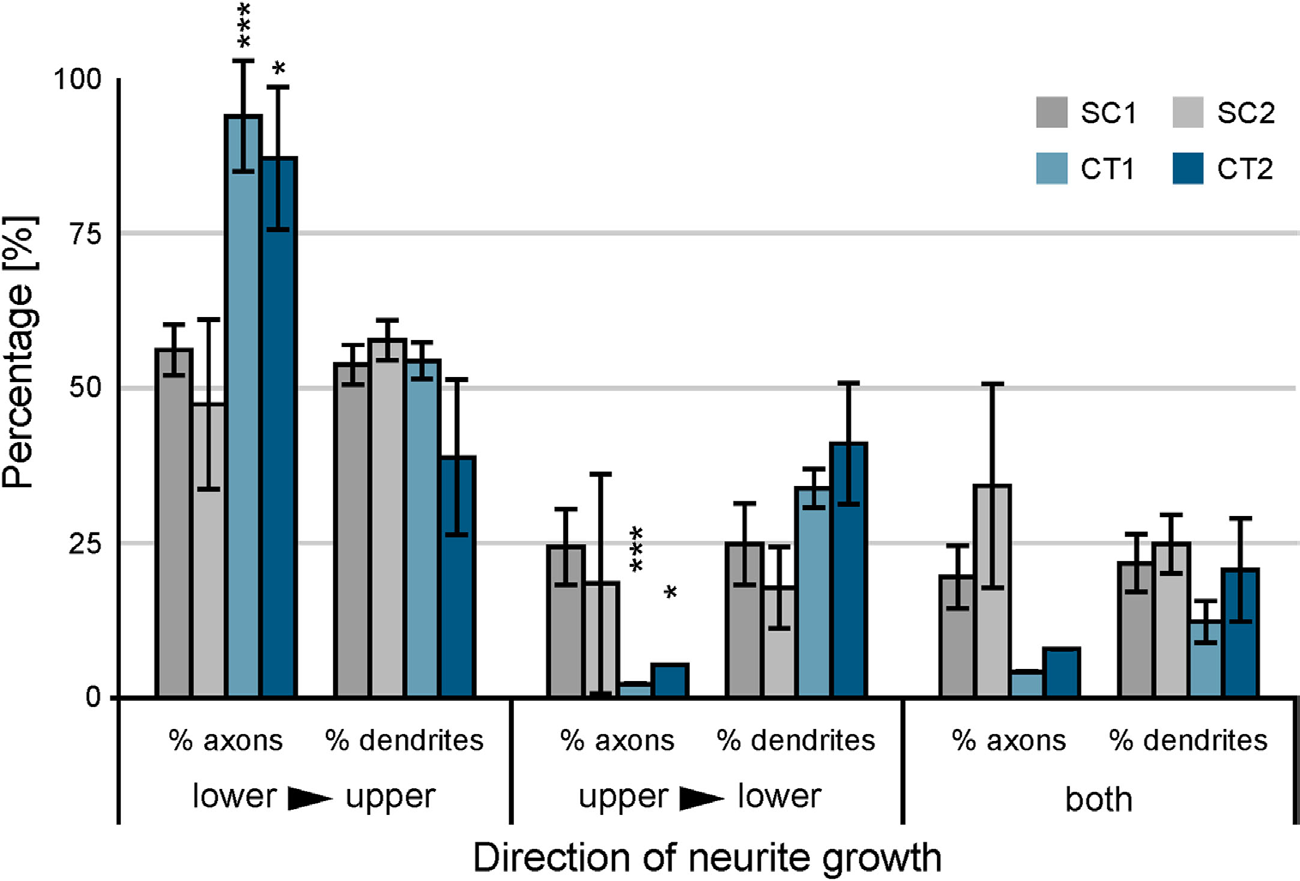
**Analysis of neuronal cell growth at the gateway between adjacent populations of neurons**. Immunofluorescence stained cultures were analyzed between 10 and 14 days *in vitro* with respect to the orientation of axonal and dendritic growth at the junction. Error bars indicate the SD. The significance of the difference between axonal and dendritic growth is calculated. ***p < 0.001, *p < 0.05.

On the SC structures, a different behavior can be observed. The funneling for the axons induced by the tip design is not as strict as observed at the CT structures. The axons still follow the protein boundaries and are guided toward the base of the upper structure, but other effects can be observed at the gateway. Axons growing at the base do not necessarily overgrow the junction and show the tendency to turn into the tip of the lower SC structure. Furthermore, not all axon bundles in the tip follow the boundary. Some turn and grow back in the opposite direction within the printed area. The statistical analysis of the growth on the gateway is shown in Figure 5. The axonal and dendritic growth was analyzed at the junction for each design. The growth across the gateway was categorized into three orientations: lower to upper structure (the intended directionality), upper to lower structure (unintended directionality), and both directions (lack of directionality). Here, the orientation of growth of the dendrite and the axon is considered irrespective of the soma from which they originate.

For both SC structures, the difference between axonal and dendritic growth within different orientations is surprisingly similar. The funnel formed by the tip guides approximately the same number of axons and dendrites crossing the lower toward upper structure. At the opposite orientation, the effect is similar. In contrast to these observations, the behavior at both CT structures shows different results (see Table 1) (neurite growth). A significant difference between axonal and dendritic growth can be observed at selected orientations. For the orientation from lower to upper structure, the percentage of axons growing along this direction distinctly exceeds the percentage of dendrites (CT1: *p* < 0.001; CT2: *p* < 0.05). The orientation from upper to lower shows an inverse behavior for axons and dendrites with the same level of significance. However, the difference between axonal and dendritic growth manifests by more than a factor of 10 in this direction. On gateways where both orientations for axons or dendrites can be observed, the difference is not as high as for the unidirectional situations.

**Table 1:** **Orientation of axonal and dendritic growth and the resulting signal propagation at the gateway**.

		Neurite growth			Signal propagation		
		% Axon	*n*	% Dendrite	*n*	% Signal	STD	*n*
SC1	Lower → upper	56.1	41	53.6	114	24.1	1.8	91
	Upper → lower	24.4	24.8	28.6	2.5
	Both	19.5	21.6	47.3	2.4
SC2	Lower → upper	47.4	38	57.6	85	28.6	12.4	7
	Upper → lower	18.4	17.7	14.3	6.2
	Both	34.2	24.7	57.1	14.3
CT1	Lower → upper	93.7	48	54.2	107	85.0	2.9	124
	Upper → lower	2.1	33.6	7.1	0.6
	Both	4.2	12.2	7.9	0.9
CT2	Lower → upper	86.8	38	38.6	44	79.2	2.3	123
	Upper → lower	5.3	40.9	6.8	0.5
	Both	7.9	20.5	14.0	0.7

As the design of the base of the SC and the CT structures is different, we also analyzed differently shaped base structures. We introduced a flat and a curved base with opposite orientation of the curvature to all four designs and characterized the growth with immunofluorescence analysis. The different designs of the base did not significantly affect the orientation of growth, neither for axons nor for dendrites, data not shown.

### Functional Analysis

**Figure 6:**
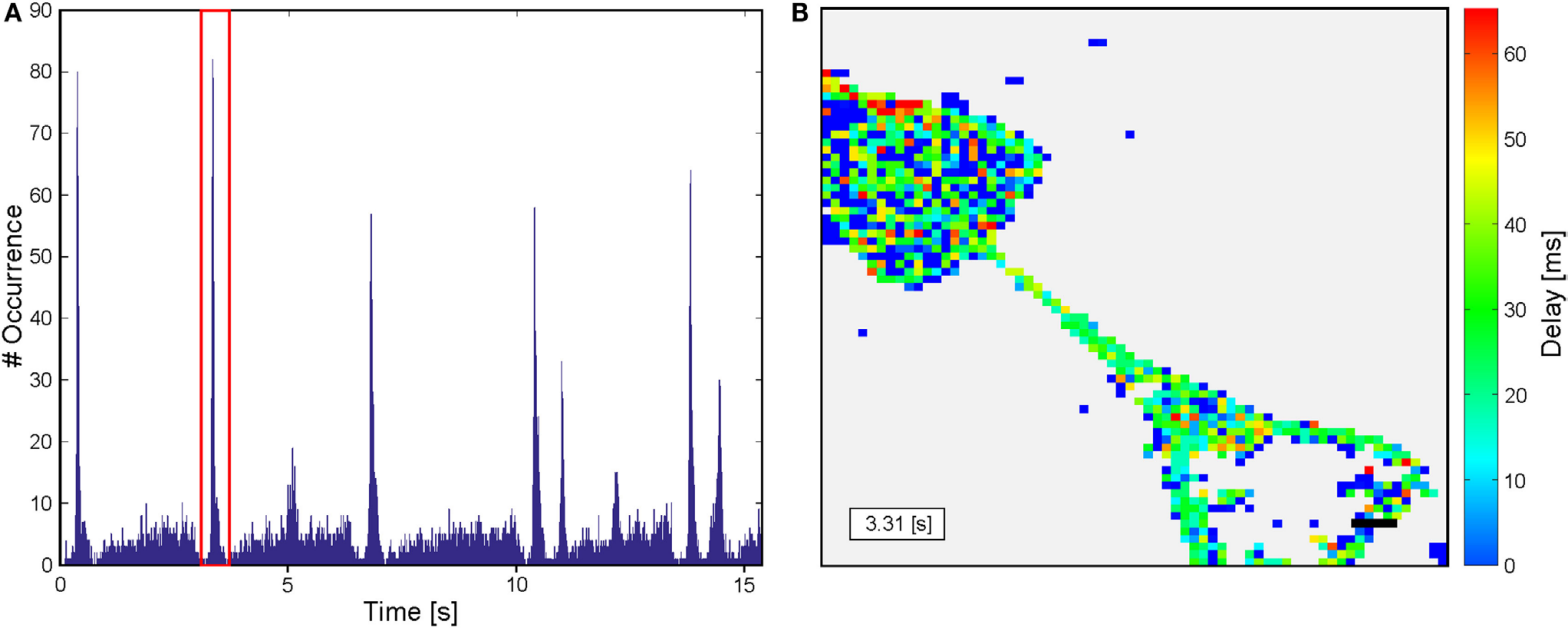
**Analysis of sequences from calcium imaging of spontaneous neuronal activity after 24 DIV**. **(A)** Histogram of occurring activity on the substrate during the recording. Substrate spanning excitations including a high number of active pixels can be clearly identified and grouped. **(B)** Delay plot of the event framed in red in the histogram. Color-coded pixels indicate the analyzed pixels, whereas the dark blue pixel did not show any activity. The differently colored pixel shows the delay of their activity related the first single event at 3.31 s of the sequence. A black scale bar is inserted at bottom right corner. Length: 50 μm.

**Figure 7:**
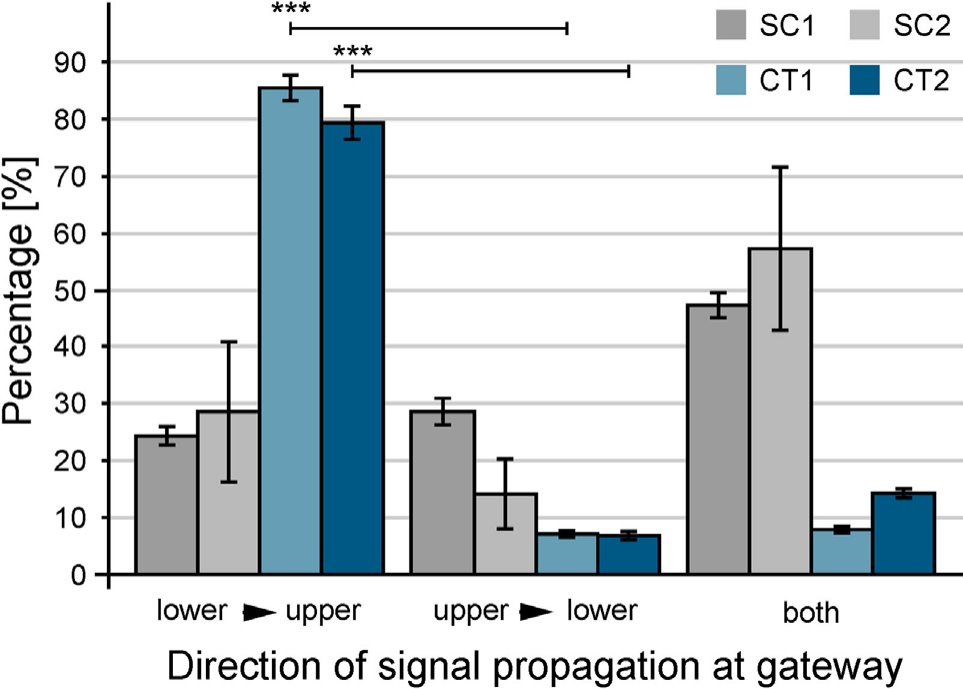
**Quantification of the guidance of signal propagation among patterned populations of neurons**. For statistical analysis, color-coded delay plots of gateways between adjacent populations of neurons were used. In total, 345 sequences of spontaneous neuronal activity were recorded between 14 and 24 days *in vitro*. Error bars indicate the SD. The orientation lower to upper is significantly preferred for CT structures in contrast to upper to lower. ***p < 0.001.

For functional analysis neurons were cultured for 14–24 days *in vitro* on structures that were shown by immunofluorescence to induce directionality of axons and dendrites. Spontaneous activity was then investigated by calcium imaging with Fluo-4 AM. Acquired sequences were analyzed with the MATLAB script described above with the aim of revealing propagation of neuronal activity on the pattern with a spatial resolution at the single-cell level and a high temporal resolution at the same time. The activity in recorded sequences was summed up resulting in histogram plots as shown in Figure 6A. These plots allow an easy separation of SSEs from background activity. For every SSE, the temporal delay between the first SE and a specific cell is visualized by a color-coded delay plot (see Figure 6B). Resulting plots of in total 345 sequences from 63 substrates and 13 preparations were analyzed manually with respect to the orientation of the propagating neuronal activity between adjacent structures. Every SSE was categorized according to the predominant orientation of the propagating signals. Figure 7 summarizes the analysis of all sequences, error bars indicate the SD. Here, both CT structures exhibit a significant (*p* < 0.001) preferential orientation of the propagating signal from the lower to the upper structure. For the CT1 design, 85% of the occurring excitations spread in this direction, while the CT2 pattern reaches a guidance of propagating signals of almost 80% (see Table 1) (signal propagation). The amount of excitations spreading in the opposite orientation (upper to lower) is significantly lower and only for a few sequences signals propagates in both directions across the gateway of the CT pattern. The situation changes completely for the SC structures. No significantly predominant orientation of propagating signals can be identified for any of these designs. The percentage of signals propagating from the lower to the upper and vice versa is almost the same (see Table 1) (signal propagation). At the same time, the amount of signals oriented in both directions exceeds both distinct orientations.

Our experiments reveal that the observed effect is independent of culture maturation. Two-thirds of the experiments were performed between 14 and 16 DIV, and a reliable spontaneous activity ensures the observation of spreading neuronal activity. The orientation of the propagating signal at the gateway is almost similar for what is shown in Figure 7. Both CT structures show a high preferential orientation of propagating singles from the lower to the upper structure with more than 85% (see Figure S3 in Supplementary Material). At the same time, the undirected orientation of signal propagation for the SC structures manifests at this time. A continuing maturation of the network does not change the situation as the last-third of the experiments reveal, which were performed between 21 and 24 DIV. The substrate-bound protein remains stable up to this time and above as it can be seen by the background fluorescence in Figure S4 in Supplementary Material. In comparison to the first experiments, the amount of signals with a preferentially orientation from lower to upper slightly decreased for the CT structures but is still significantly larger than any of the other two orientations (*p* < 0.01). As a consequence, the presented results refer to the summarized data from all recorded sequences.

Independent from the orientation of signal propagation at the gateway, a critical number of cells could be determined that is required for synchronized spontaneous activity. By reducing the surface area from CT1 to CT2, the total number of cells could be reduced without a reduction of the cell density. This way a critical cell density of 150 cells/mm^2^ was obtained, which corresponds to ~10 cells per CT2 structure. A further decrease of cell density results in an unsynchronized firing pattern, which strongly impacts the signal propagation and excitation among populations of neurons. No impact on signal propagation speed could be found for any of the investigated structures. Color-coded delay plots were analyzed to estimate the time to excite signaling cascades across the pattern. As the dimensions of the investigated area are known, also the direct distance between starting and final point of a signal cascade is known. In our analysis, only excitations that disperse across the whole frame and pass a junction were included. We calculated a propagation velocity of 13 ± 3 μm/ms (velocity ± SD) at 24°C RT, irrespective of pattern design and orientation of the spreading excitation.

## Discussion

In this study, we present a microstructuring method enabling exclusive cell growth on structured protein patterns. As a consequence, populations of neurons can be shaped by predetermined protein structures with defined gateways. The design of the structures highly influences the neuronal outgrowth, whereas the impact on axonal and dendritic growth differs with chosen geometric parameters. At the CT structures, the funnel-like shaped tip leads to a highly selective growth at the gateway shown in Figure 4. All axons within the bundle growing in the funnel of the lower structure sprout into the upper pattern, where the bundle roves to form connections with the adjacent population. On the other side of the gateway, the situation looks different; here, the internal tension within the axon results in a bundle growth that passes the gateway without noticeable response. Here, the prior orientation to the cytoskeleton impacts the orientation of the axonal outgrowth by the tension generated by microfilaments inside the axon (Baas and Ahmad, 2001; O’Toole et al., 2008, 2015; Suter and Miller, 2011; Roth et al., 2012). A harsh turn of the bundle into the lower structure would cause a reorientation and rearrangement of the internal compartments of the axon what is energetically unfavorable. For the dendritic growth at the CT structures, guidance effect is not as evidenced as for axons. Here, the lower stiffness and higher affinity for branching allow the dendrites to enter the funnel from both sides. Even though, a preferential orientation of dendritic growth is favored to a growth in both orientations at the gateway at the same time. For the pie slice-like SC structures, a clear difference between the growth of axons and dendrite cannot be recognized in either direction. The apex angle of the lower structure is too large such that the funneling effect for axons and dendrites was equivalent. Considering the internal tension within the axon and the resulting predetermined orientation of its growth a turn form the upper to the lower structure is more likely to occur than for the CT structures. For any orientation of growth on the pattern, the amount of axons balances the amount of dendrites growing in this distinct orientation for the SC structures.

As a consequence, we conclude that an explicit difference between the orientation of growth of axons and dendrites has to be established to induce a functional polarity at the gateway of adjacent structures. Furthermore, it is evident that signal directionality is dominated by the ability to control orientation of axonal outgrowth more than by the ability to direct dendritic growth. This separation of axonal and dendritic growth can be achieved by a funnel-like tip with an apex angle of 6°. If this apex angle would be applied to the SC structures, the total cell adhesive area of the SC structures would be reduced by a factor of 10. Therefore, the CT design with a funnel-like tip with an apex angle below 6° and an increased area for the subnetwork caused by the curved flanks offers the option to grow a highly ramified population of neurons that connects to a neighboring population with a predefined functional directionality. Another reason to avoid narrow structures is the internal tension in the axon (Roth et al., 2012) that results in a network tension by itself. We identified this network tension to be responsible for the distinct gab between the boundary of the protein and the axon bundle. The emerging tension pulls the bundles toward the center of the population at which the bundles cannot oppose as they do not have a balancing tension from the direction.

The conduction velocity measured in this study is an order of magnitude lower than reported values from *in vivo* studies (Garwicz and Andersson, 1992) and cerebellar slices (Vranesic et al., 1994; Dellal et al., 2012). While these studies were performed *in vivo* at 36°C or at slices at 32–35°C and an atmosphere of 95% O_2_ and 5% CO_2_, our experimental conditions differ drastically. Our trials were performed at 24°C in *in vitro* cultures with a low amount of astrocytes and glial cells supporting neuronal activity. The temperature difference itself causes a nerve conduction velocity decrease by a factor of 2–3 following the Arrhenius equation. Additionally, the exact trajectory of the dispersing signal is unknown in our network, and we cannot report the exact distance the dispersing excitation covered. Furthermore, our value includes synaptic delay ranging from 1 to 5 ms (Kandel et al., 2000) per synapse of an unknown number of synapses involved in the trajectory. Thus, we believe that our reported value of 13 μm/ms is a reasonable velocity for an excitation dispersing in a neuronal network under these conditions and corresponds to reported values in literature (Orlandi et al., 2013).

With this work, we aim at engineering and characterizing microstructured neuronal networks in order to generate highly predictive *in vitro* neuronal circuitries, which can be used to investigate network activity by electrical or optical means as well as allowing predictive pharmacology, disease modeling, and learning processes. Another possible application for the presented system could be the investigation of network activity and connectivity in the aftermath of axonal and dendritic regeneration after thermal (Rinklin et al., 2015) or mechanical lesion.

## Author Contributions

JA has designed the study, has acquired, analyzed, and interpreted data, and he has written the article. AO has conceived and designed the study, has interpreted data, and has written the manuscript.

## Conflict of Interest Statement

The authors declare that the research was conducted in the absence of any commercial or financial relationships that could be construed as a potential conflict of interest.
