# Supplementary material for: Signal Propagation between Neuronal Populations Controlled by Micropatterning
Source: Front Bioeng Biotechnol. 2016 Jun 15;4:46. doi: 10.3389/fbioe.2016.00046 (PMC4908115; doi:10.3389/fbioe.2016.00046)
Supplement: Supplementary file 1 [file data_sheet_1.docx]

Supplementary Material

Signal propagation between neuronal populations controlled by micropatterning

Jonas Albers1, Andreas Offenhäusser2*

*** * Correspondence:** Prof. Andreas Offenhäusser, Peter Grünberg Institute/Institute of Complex Systems, Bioelectronics (PGI-8/ICS-8), Forschungszentrum Jülich GmbH, D-52425 Jülich, Germany

a.offenhaeusser@fz-juelich.de

# Supplementary Figures


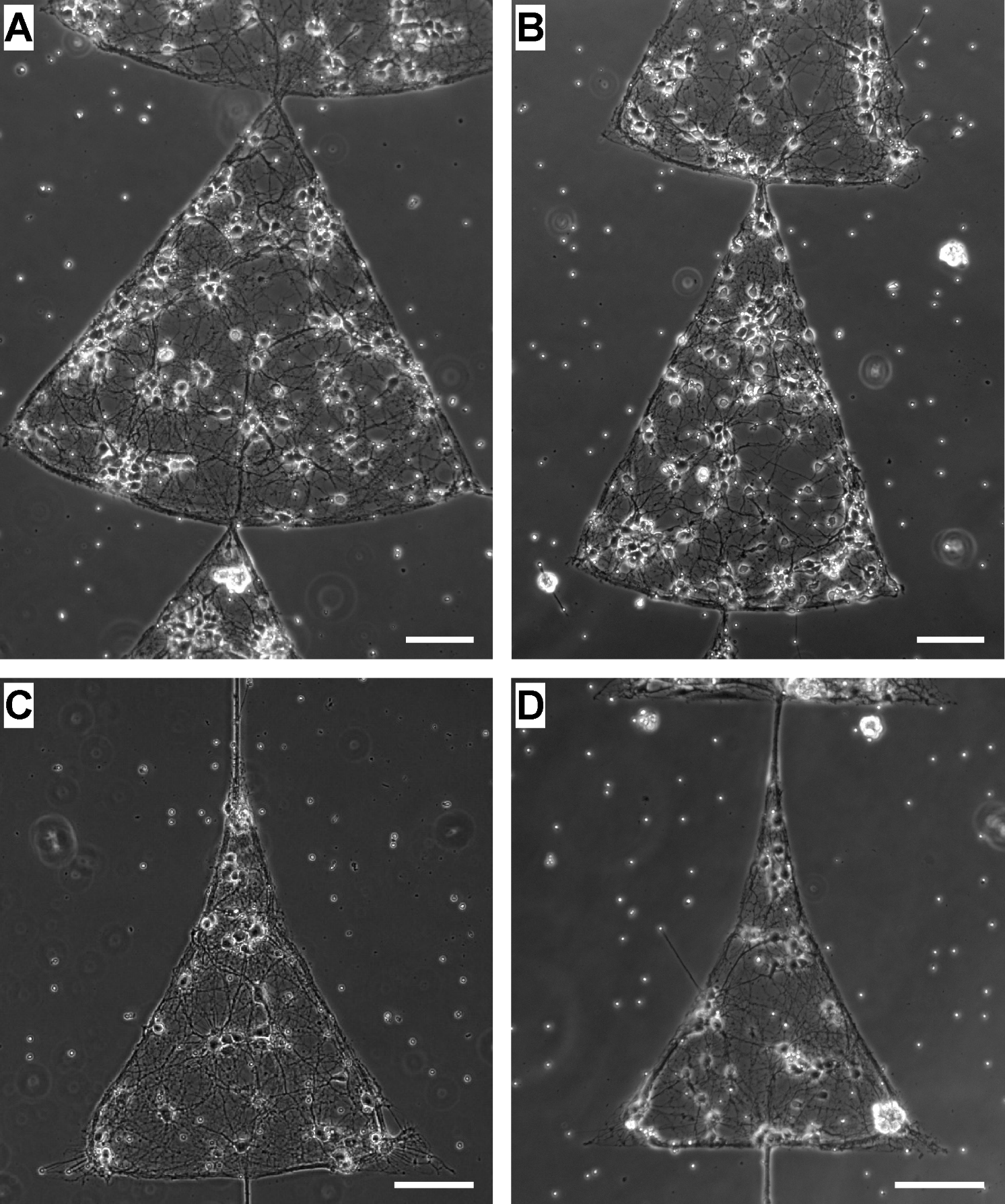


**Supplementary Figure 1.** Cell growth on the different investigated structures after 7 DIV. The neurons on all structures form a ramified network covering the entire structures. Along the edge of the pattern a bundle of neurites forms that surrounds the population. (**A**) SC1 structure, (**B**) SC2 structure, (**C**) CT1 structure, (**D**) CT2 structure. Scale bar: 100 µm.

**
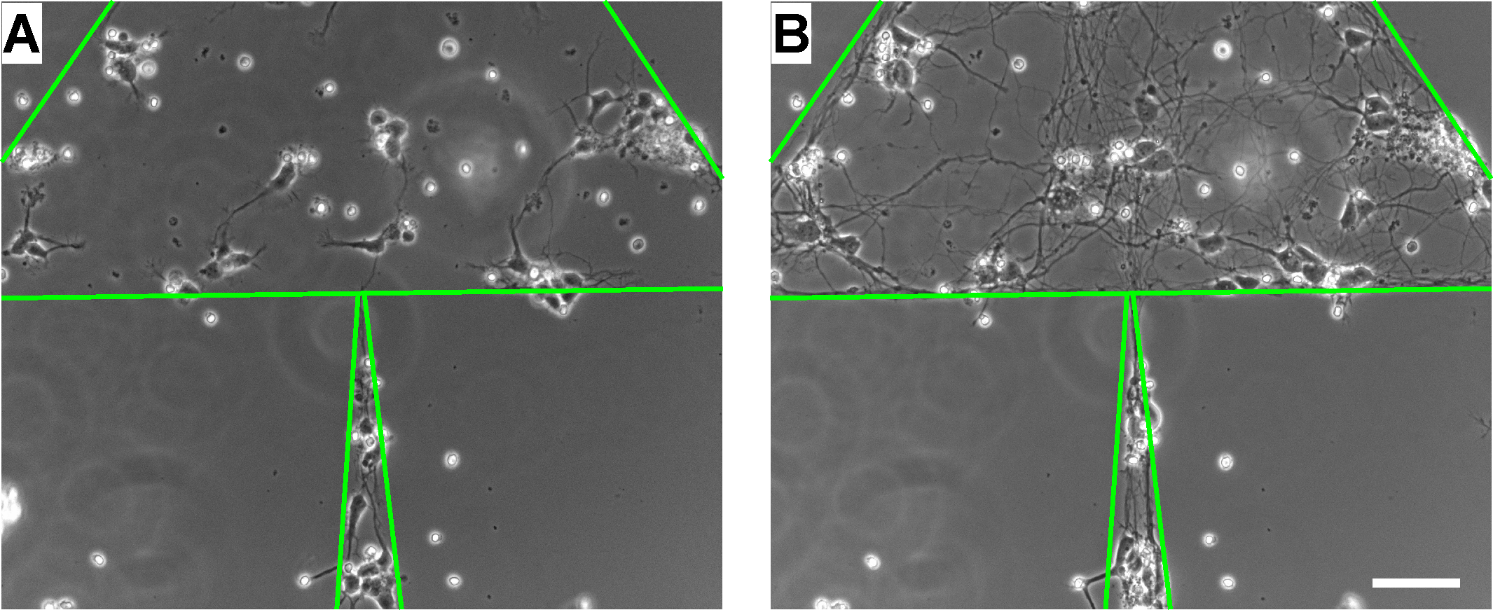
**

**Supplementary Figure 2.** Live cell imaging experiments on patterned neuronal populations reveal that the neurites follow the pattern whereas a ramified network is formed within the pattern. The green lines indicate the edge of the CT1 structure that is reconstructed from an overlaid fluorescence image from the FITC labeled PLL. (A) Cell growth after 1 DIV. Axons and dendrites started to sprout and single neurites can be tracked to individual cells. (B) Cell growth after 4 DIV. The cells grow within the printed protein pattern. At most of the edges the neurites grow along the passage between the coated and uncoated surface. Within the pattern a ramified network starts to form involving a high number of neurites that make it impossible to associate a single neurite to a soma.

**
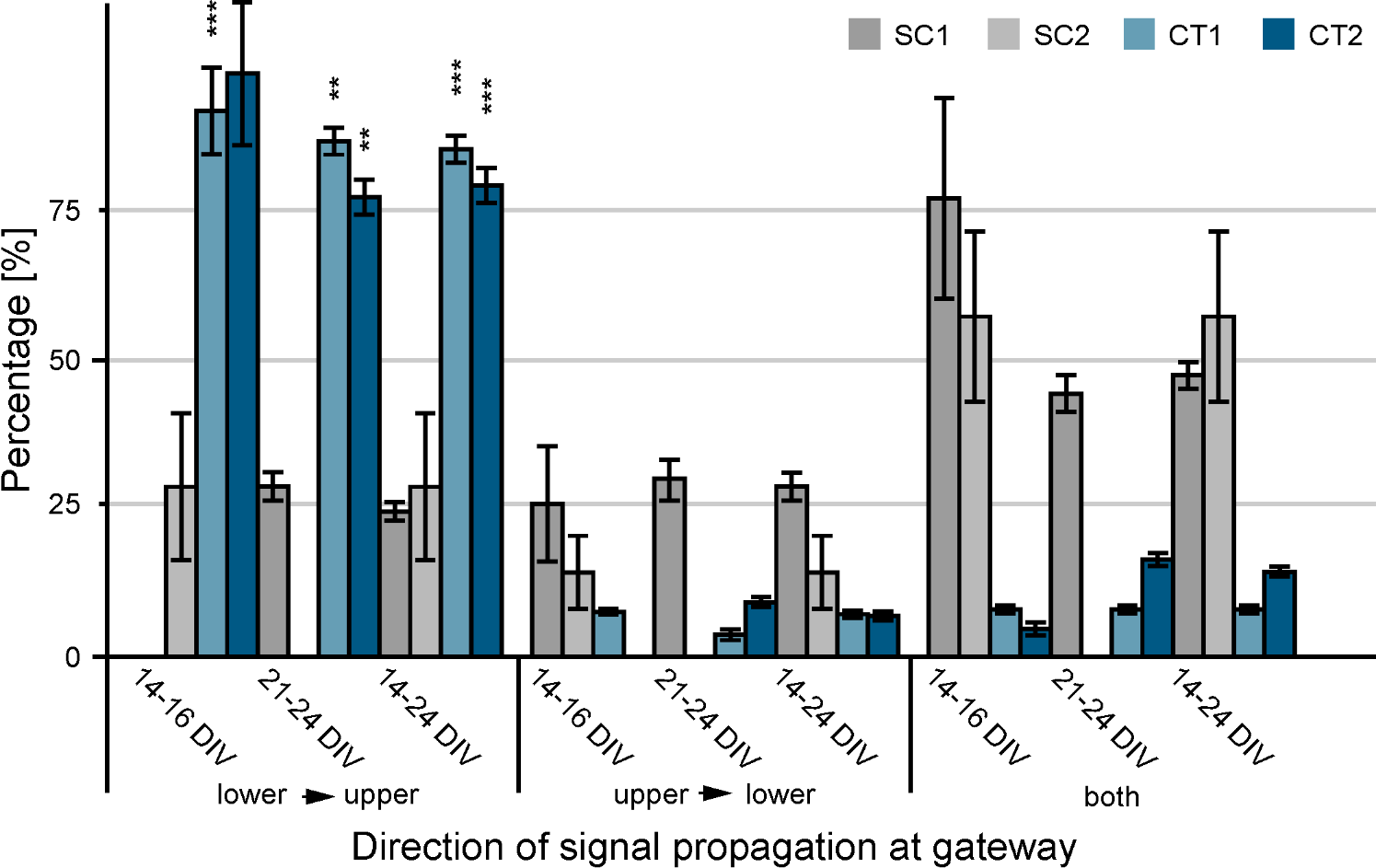
**

**Supplementary Figure 3.** Orientation of signal propagation at the gateway at different points in time. Two third of the recorded sequences were obtained in experiments after 14-16 DIV and a clear preferential orientation for CT1 and CT2 can be seen. Here the stated significance is the lowest significance that is found for all three orientations. The SC structures in contrast do not exhibit a dominant network polarity. To see if an increase in network maturation impacts the functional polarity a second set of experiments was performed after 21 and 24 DIV. No drastic changes in functional polarity of the network can be observed. As a consequence the results from both sets of experiments are presented in summary. Error bars indicate the standard deviation. ***: p<0.001, ** p<0.01


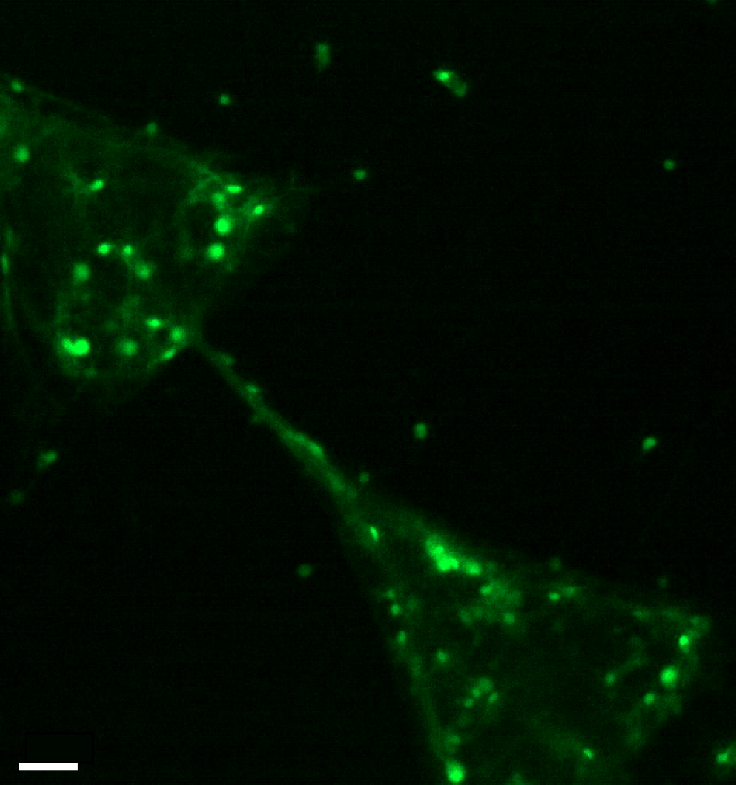


**Supplementary Figure 4.** A frame from a video sequence of a calcium imaging experiment after 21 DIV reveals that the FITC labeled PLL is still visible after that time. As a consequence this means that the protein is present after that time and provides cell adhesion sided. Scale bar 50 µm.
